# Updates on Tinea Incognita: Literature review 

**DOI:** 10.22034/cmm.2023.345069.1425

**Published:** 2023-06

**Authors:** Aliasghar Ghaderi, Pegah Tamimi, Alireza Firooz, Maryam Fattahi, Mona Ghazanfari, Mahsa Fattahi

**Affiliations:** 1 Center for Research and Training in Skin Diseases and Leprosy, Tehran University of Medical Sciences, Tehran, Iran; 2 Mohebe Kowsar Hospital, Tehran, Iran; 3 Invasive Fungi Research Center, Communicable Diseases Research Institute, Mazandaran University of Medical Sciences, Sari, Iran; 4 Immunology, Asthma and Allergy Research Institute, Tehran University of Medical Sciences, Tehran, Iran; 5 Children’s Medical Center, Pediatric Center of Excellence, Tehran University of Medical Sciences, Tehran, Iran

**Keywords:** Dermatophytes species, Immunosuppressive medications, Tinea incognita, Topical corticosteroids

## Abstract

**Background and Purpose:**

Tinea incognita (TI), or the other equivalent tinea atypica, is a term used to declare the atypical presentation of dermatophyte infections caused by the administration of steroids or other immunosuppressive medications which modulate the local and systemic immune response. It can mimic other dermatoses; hence making diagnostic challenges for dermatologists. Tina incognita may be misdiagnosed as many dermatoses. Based on previous studies, corticosteroids may cause different clinical manifestations of dermatophytes that might be very different from those that are commonly described.

**Materials and Methods:**

This narrative review was conducted using PubMed and Scopus databases. Search terms included “Tinea incognita” and “Atypical dermatophytosis”. The search strategy included meta-analyses, randomized controlled trials, clinical trials, observational studies, reviews, and case reports. The search was restricted to articles written in the English language from 2006 to Feb 01, 2023. Moreover, duplicate articles and non-available full-text articles were excluded. The extracted data of the search results were retrieved in this study. The morphological patterns, prevalence, sight of infection, and causative agents were also described.

**Results:**

Prevalence of different patterns of TI were recorded as 50% (431 out of 862 cases) for eczema-like lesions followed by psoriasis-like and 6.61% (57 out of 862) for parapsoriasis-like pattern. Moreover, each of the rosacea-like and pyoderma-like lesions equally accounted for 4.98 % of cases (43 out of 862). In addition, the prevalence of causative agents was reported as follows: *Trichophyton rubrum* accounted for 247 isolates (40%) as the most prevalent, followed by *Trichophyton mentagrophytes* (n=152, 24%) and *Microsporum canis* (n=119, 19%).

**Conclusion:**

Tinea incognita is a great mimicker; hence, dermatologists should obtain a full medical history of the patients to make correct diagnoses. It is vital to encourage an exact identification of the etiological agent according to the internal transcribed spacer sequencing in some uncertain cases. This review highlights the importance of mycological tests and fast diagnosis of TI, especially in cases of atypical skin lesions, to choose appropriate treatment and avoid the spread of drug-resistant species.

## Introduction

Dermatophytes are a group of fungi that cause skin infections known as dermatophytosis, ringworm, or tinea [ 1 ]. Dermatophytes are keratinophilic and keratinolytic fungi that colonize keratinized tissues, like skin, hair, and nails, and invade the stratum cornea of these tissues [ 1 ]. This group of fungi belongs to the genera *Trichophyton*, *Epidermophyton*, *Nannizzia*, *Paraphyton*, *Lophophyton*, *Microsporum*, and *Arthroderma* in dermatophytes [ 2 ]. 

Susceptibility to dermatophytosis depends on several risk factors, like wet climate and geographical region, contact with animals, underlying diseases, and age. An optimal environment enables some special species to grow. Tinea incognito (TI) is a term first stated by IVE and Marks in 1968 to declare the condition caused by the use of steroids, leading to an atypical presentation of dermatophyte infections [ 3 ]. The term “tinea atypica” can sometimes be used instead of TI. The term TI is precisely used for drug-stimulated conditions, but atypical tinea appears in immunocompetent individuals who are not using any topical and systemic immunosuppressive drugs [ 4 ]. However, there is a viewpoint that the phrase “tinea incognito” is grammatically wrong and it is better to use “tinea incognita” instead [ 5 ]. 

The TI is mostly a side effect of abusing topical or systemic corticosteroids. It is usually a diagnostic challenge for dermatologists as this infection does not have the classic presentations and can imitate other illnesses since corticosteroids can mask signs of underlying lesions [ 6 ]. Immunomodulators, such as calcineurin inhibitors (including tacrolimus and pimecrolimus) or tumor necrosis factor inhibitor treatment [ 7 ], can also cause TI, but it is less commonly observed than corticosteroids [ 8 ]. There are reports of other drugs, such as fumaric acid esters (a drug used as systemic antipsoriatic) that may cause TI [ 9 ]. The TI cases have had a worldwide increase, and delays in diagnosis and proper treatment may lead to generalized tinea or leave permanent marks on the patient.

Topical corticosteroids can disrupt the local immunity system and the infection may become widespread [ 6 ]. This mechanism starts with the production of lipocortin following the binding of steroids to their cytoplasmic receptors in keratinocytes. Lipocortins disrupt inflammatory mediators and arachidonic acid production and prevent the migration of polymorphonuclear cells. Lipocartin can also increase vasoconstriction and decrease vascular permeability, which leads to edema and erythema reduction [ 8 ]. 

The increase in coinfections, TI, and geographic distribution decreases the chance of treatment success, indicating the need for highly efficient diagnostic approaches. The same concern exists regarding the increased incidence of recurrent and hard-to-treat dermatophytosis. 

## Materials and Methods

### Search strategy

This narrative review was performed using PubMed and Scopus databases. Search terms included “Tinea incognita” and “Atypical dermatophytosis”. The search strategy included meta-analyses, randomized controlled trials, clinical trials, observational studies, reviews, and case reports. The search was restricted to articles written in the English language from the beginning of 2006 to Feb. 01, 2023. Moreover, duplicate articles and non-available full-text articles were excluded. The extracted data of the search results were retrieved in this study. The morphological patterns, prevalence, sight of infection, and causative agents were also described.

### Updates on the epidemiology of tinea incognita

Tinea incognita does not seem to be a common disease. The exact number of cases is not known, but it is said that TI forms less than 1% of total dermatophytes in the world [ 1 ]. However, there are an increasing number of reports of atypical presentations and steroid-modified tinea. Diverse species, which are causative agents of TI, are distributed into three subspecies: zoophilic (*Trichophyton verrucosum*, *Trichophyton mentagrophytes* [ 10 ], and *Microsporum canis*), geophilic (*Microsporum gypseum*, *Microsporum ferrugineum*, and *Microsporum langeronii*), and Anthropophilic (*Trichophyton interdigitale*, *Epidermophyton floccosum*, *Trichophyton rubrum*, and *Trichophyton violaceum*) [ 11 ]. 

**Figure 1:**
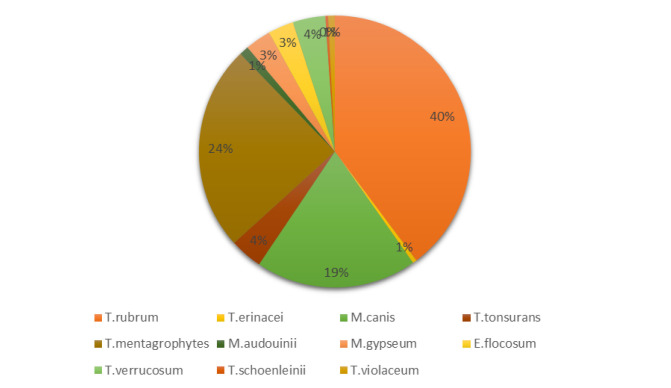
Causative agents of Tinea incognita in previous studies

The frequency of pathogenic dermatophyte species in available literature from 2006 to Feb. 01, 2023 (Table 1, Figure 1) indicated that a total of 614 isolates affiliated with 11 species were detected as causative agents of TI in 26 reviewed studies. These species were *T. rubrum* (n=247, 40.22%), *T. mentagrophytes* (n=152, 24.75%), *M. canis* (n=119, 19.38%), *T. verrucosum* (n=24, 3.9%), *Trichophyton tonsurans* (n=24, 3.9%), *E. floccosum* (n=19, 3.09%), *M. gypseum* (n=19, 3.09%), *Microsporum audouinii* (n=7, 1.14%), *T. violaceum* (n=5, 0.81%), *Trichophyton erinacei* (n=3, 0.48%), *Trichophyton Schoenleinii* (n=2, 0.32%).

**Table 1:** Anatomical site distribution, different patterns of tinea incognita and etiological agents.

Year	Study	Pattern of lesion	Site of lesion	Prevalence	Culture Result/n	Age/ Gender
2023	Hu et al.[ 70 ]	Eczema-like	Ear	1	*T. rubrum* (1)	32/F
2022	Babakoohi et al. [ 71 ]	Cutaneous T-cell Lymphoma-like	Shin (1)	1	ND	62/F
	Zhi et al.[ 72 ]	Eczema-like	Ear	1	*M. canis* (1)	4/M
	Bhagyashree et al.[ 25 ]	Pseudoimbricata (Indecisive)	Trunk, Groin, Face	42	*T. tonsurans* (18), *T. mentagrophytes* (14), *M. audouinii* (7), *T. rubrum* (3)	24.57* / M:2F
	Cunningham et al.[ 15 ]	Allergic contact dermatitis-like	Face (3)	3	*T. mentagrophytes* (3)	6/F
						3/F
						10/F
2021	Starace et al.[ 51 ]	Eczema-like (pediatric)	Trunk (3), Leg (2), Back (2), face (1)	8	*M. gypseum* (8)	2/F
						5/M
						4/M
						8/F
						5/F
						4/F
						7/M
						6/M
	Eichhoff et al. [ 7 ]	Pusular psoriasis like	Trunk, Extremities	1	*T. rubrum* (1)	46/M
2020	Kalkan et al.[ 19 ]	Subcorneal pustular dermatosis- like	Trunk	1	*Dermatophyte* spp.	89/F
	Frantz et al.[ 73 ]	Contact dermatitis-like	Hand (1)	1	*T. mentagrophytes* (1)	37/F
2019	Henry et al. [ 31 ]	Extensive form of Tinea corporis	Whole body	1	*T. mentagrophytes* (1)	45/F
2018	Lammoglia-Ordiales et al.[ 20 ]	Majocci granuloma	Hand	1	*Trichophyton erinacei* (1)	31/M
	Gathings et al. [ 74 ]	Allergy-like and tattoo-associated	Calf ( tattoo site)	1	ND	52/M
2016	Jowkar et al. [ 17 ]	Dermatitis herpetiform-like	Trunk Upper extremities face	1	Dermatophyte spp.	57/F
	Dutta et al. [ 34 ]	Eczema-like Nummular eczema	Arm(3), Extremities(1), Trunk(1), Leg(3), Hand(1)	9	*T. rubrum* (4), *T. mentagrophytes* (1), *E. floccosum* (1)	28.47*/54M
						46F
		Atopic eczema	Face (8)	8	*T. rubrum* (3), *T. tonsurans* (1)
		Discoid lupus erythematosus (DLE)-like	Face (2)	2	*T. mentagrophytes* (1)	
		Psoriasis-like	Palm (4), Feet (3)	6	*T. rubrum* (3), *T. mentagrophytes* (1)	
		Rosacea like	Face (5)	6	*T. rubrum* (1), *T. mentagrophytes* (1)	
		Seborrheic dermatitis-like	Face (4), Trunk (5)	9	*T. rubrum* (2), *T. mentagrophytes* (7)	
		Systemic lupus erythematosus (SLE)-like	Face (2)	2	*T. mentagrophytes* (1)	
		Perioral dermatitis-like	Face (2)	2	*T. rubrum* (1)	
		Malar rash	Face (10)	10	*T. rubrum* (3)	
		Photosensitive rash	Face (8)	6	*T. rubrum* (ND), *T. mentagrophytes* (3)	
		Hensens’ disease	Face (5), Leg(1)	6	*T. rubrum* (3)	
		Large annular lesion	Arm (2), Trunk (1), Groin (1), Leg (1)	5	*T. rubrum* (2), *T. mentagrophytes* (2)	
		Airborne contact dermatitis	Face (3), Generalized (1), Trunk (2)	4	*T. rubrum* (2), *T. tonsurans* (1)	
		Ichthyosis	Trunk (4)	4	*T. mentagrophytes* (1)	
		Irritant dermatitis	Face (3), Trunk (1)	4	*T. rubrum* (3)	
		Striae	Legs (2), Arm (1), Groin (1)	4	*T. rubrum* (1), *T. mentagrophytes* (3)	
		Granuloma annulare	Hands (2), Arm (1), Legs (1)	4	*T. rubrum* (1), *T. mentagrophytes* (1)	
		Maculopapular rash	Trunk (2), Face (1)	2	*T. rubrum* (1)	
		Pityriasis rosea	Trunk (2), Leg (1), Groin (1)	2	*T. rubrum* (1)	
		Folliculitis	Scalp (2)	2	*T. tonsurans* (1), *M. canis* (1)	
		Erythema multiforme	Trunk (1)	1	*T. mentagrophytes* (1)	
		Vermilion dermatitis	Face (1)	1	*T. mentagrophytes* (1)	
		Ecchymosis	Trunk	1	*T. rubrum* (1), *E . floccosum* (1)	
		Depigmentation with scaling or scaly achromic patches or vitiligo-like	Leg	1	*T. rubrum* (1)	
2014	Tan et al.[ 22 ]	syphilis-like	Palm, Soles, penis	1	*T. rubrum* (1)	56/M
	Park et al.[ 75 ]	Herpes simples-like	Face	1	Dermatophyte spp.	32/M
2013	Calcaterra et al.[ 27 ]	Rosacea-like	Face	1	*T. mentagrophytes* var. mentagrophytes (1)	47/F
	Kim et al.[ 42 ]	Eczema-like	Face(53), Trunk (68), Groin(25), Hand(22), Foot(37), Multiple region (27)	232	*T. rubrum* (49), *T. mentagrophytes* (6), *M. canis* (6), *T. tonsurans* (2), *T. verrucosum* (2), *M. gypseum* (2)	44*/125F
						158M
		Impetigo-like	Face(2), Trunk(2)	4		
		Psoriasis-like	Face (1), Trunk(9), Groin (1), Multiple region (6)	17		
		Seborrheic dermatitis-like	Face(3), Trunk(1)	4		
		SLE-like	Face(6), Multiple region (1)	7		
		Folliculitis	Head (1), Multiple region (1)	2		
		Depigmentation with scaling or scaly achromic patches or vitiligo-like	Face (2)	2		
		Urticarial-like	Face (1), Trunk (1), Groin (1)	3		
		Lichen simplex chronic-like or Lichen ruber planus- like	Trunk (1), Multiple region (1)	2		
		Xerosis- like	Foot (2)	2		
		Other	Face (3), Trunk (5), Groin (1), Multiple region (3)	12		
Zisova et al.[ 11 ]	Pyoderma	Face, Pubic	2	*T. mentagrophytes* (1), *M. canis* (1)	53/M
					23/F
					46/M
					15/M
		Herpes zoster	Face	1	*T. rubrum* (1)	62/F
		Impetiginized eczema	Calf	1	*T. verrucosum* (1)	48/M
		Tuberculum Mulgentium-like	Hand	1	*T. verrucosum* (1)	48/F
		Psoriasis-like and seborrheic dermatitis-like	Face	1	*T. rubrum* (1)	78/F
		Folliculitis	Neck	1	*T. mentagrophytes* (1)	74/F
		Pemphigoid-like	Genitalia	1	*T. rubrum* (1)	
	Amano et al.[ 18 ]	Pemphihus erythematous-like	Neck, Face	1	*M. canis* (1)	
2012	Atzori et al.[ 16 ]	Eczema-like	Trunk (26), Limbs (25), Face (8), Abdomen (6), Buttock (5)	70	*M. canis* (30), *T. rubrum* (19), *T. mentagrophytes* var. *mentagrophytes* (13), *T. interdigitale* (6), *E. floccosum* (2).	ND (6 children)/ 71M
						83F
		Impetico-like	Trunk (14), Limbs (8), Face (3)	25	*M. canis* (11), *T. rubrum* (2), *T. mentagrophytes* var. *mentagrophytes* (10), *M. gypseum* (2)	
		DLE-like	Face (9), Chest (3)	12	*M. canis* (6), *T. rubrum* (4), *T. mentagrophytes* var. *mentagrophytes* (2)	
		Polymorphous light eruption-like	Face (9), Chest (2)	11	*M. canis* (7), *T. rubrum* (4)	
		Psoriasis-like	Trunk (6), Limbs (5)	11	*M. canis* (3), *T. rubrum* (6), *T. mentagrophytes* var. *mentagrophytes* (2)	
		Rosacea-like	Face (8)	8	*M. canis* (2), *T. rubrum* (4), *T. mentagrophytes* var. *mentagrophytes* (2)	
		Seborrheic dermatitis-like	Face (6), Chest (1)	7	*M. canis* (2), *T. rubrum* (5)	
		SLE-like	Face (2), Chest (2)	4	*M. canis* (2), *T. rubrum* (2)	
		Herpes simplex-like	Face (2)	2	*M. canis* (2)	
		Herpes zoster-like	Trunk	1	*M. canis* (1)	
		Perioral dermatitis-like	Face	1	*M. canis* (1)	
		Pyoderma gangrenosum-like	Leg	1	*M. gypseum* (1)	
		Burn-like	Hand	1	*T. verrucosum* (1)	
	Zarei Mahmoudabadi et al.[ 76 ]	Psoriasis-like	Groin	1	*E. floccosum* (1)	25/M
	Mansouri et al. [ 21 ]	Parapsoriasis-like	Trunk, Upper & lower extremities	1	*T. Schoenleinii* (1)	80/F
	Ishizaki et al.[ 77 ]	Allergy-like & tattoo associated	Face (tattoo site)	1	*M. gypseum*(1)	63/F
2011	Ansar et al.[ 38 ]	Eczema-like	Trunk (6), Hand (6)	12	*T. rubrum* (3), *T. mentagrohytes*. var *mentagrohytes* (3), *E. floccosum* (1), *M. canis* (1), *T. mentagrohytes*. var *interdigitale* (3), *T. verrucosum* (3),	32.6*/29M
						27F
		Rosacea-like	Face (9)	9	*T. verrucosum* (3), *T. rubrum* (2), *T. mentagrohytes*. var *mentagrohytes* (2), *M. canis* (1), *T. mentagrohytes*. var *interdigitale* (1)	
		Seborrheic dermatitis-like	Face (4), Groin (3)	7	*T. mentagrohytes*. var *mentagrohytes* (1), *E. floccosum* (1), *M. canis* (1), *T. mentagrohytes*. var *interdigitale* (2), *T. verrucosum* (2)	
		Pyoderma-like	Trunk (5), Hand (1)	6	*T. mentagrohytes*. var *mentagrohytes* (2), *T. verrucosum* (4)	
		Pytiriasis rosea-like	Trunk	5	*E. floccosum* (1), *M. canis* (1), *T. mentagrohytes*. var *interdigitale* (1), *T. verrucosum* (1), *T. violaceum* (1)	
		Psoriasis-like	Foot	3	*E. floccosum* (1), *T. rubrum* (1), *T. verrucosum* (1)	
		Allergic-like	Groin	3	*E. floccosum* (1), *M. canis* (1), *T. verrucosum* (1)	
		Contact dermatitis-like	Foot	2	*E. floccosum* (1), *T. rubrum* (1)	
		Petaloid form (contact dermatitis- seborrheic -like)	Trunk	2	*T. verrucosum* (1), *T. mentagrohytes*. var *interdigitale* (1)	
		Inverse psoriasis	Groin	2	*T. verrucosum* (1), *T. mentagrohytes*. var *interdigitale* (1)	
		Folliculitis	Scalp	2	*T. verrucosum* (1), *T. Schoenleinii* (1)	
		Photosensitivity-like dermatitis	Face	1	*T. verrucosum* (1)	
		Alopecia	Scalp	1	*T. violaceum* (1)	
		DLE-like	Face	1	*T. mentagrohytes*. var *mentagrohytes* (1)	
	Satana et al. [ 26 ]	Allergy-like	Face	1	*T. mentagrophytes* complex (1)	17/F
2010	Lange et al. [ 14 ]	Zooster-like	Face	1	*T. mentagrophytes* (1)	54/F
2009	Kaštelan et al. [ 30 ]	Multiple nummular scalu papules and plaques	Trunk, Upper extremities	1	*T. rubrum* (1)	72/F
2007	Sanchez-castellanos et al. [ 78 ]	Erythematous scaly plaques	Face	1	*T. mentagrophytes* var. *mentagrophytes* (1)	2/F
	Nenoff et al. [ 45 ]	Rosacea-like And Erythematous scraches	Face (2)	2	*T. rubrum* (2)	58/F
						64/M
2006	Romano et al. [ 79 ]	Eczema-like	Face (33), Trunk (26), Limbs (23), Other (2)	89	*T. rubrum* (51), *T. mentagrohytes*. var *interdigitale* (18), *T. mentagrohytes*. var *mentagrohytes* (5), *T. violaceum* (3), *E. floccosum* (3), *M. canis* (7), *M. gypseum* (2)	42*/ 102F
						98M
		DLE-like	Face (9), Hand (2)	15	*T. rubrum* (11), *T. menatgrophytes* (3), *E. floccosum* (1)	
		Psoriasis-like	Trunk (7)m, Limbs (6)	13	*T. rubrum* (9), *T. mentagrohytes*. var *mentagrohytes* (1), *M. canis* (1), *M. gypseum* (1), *E. floccosum* (1)	
		Rosacea like	Face	17	*T. rubrum* (12), *T. mentagrohytes*. var *interdigitale* (1), *T. mentagrohytes*. var *mentagrohytes* (1), *M. canis* (3)	
		Seborrheic dermatitis-like	Face (2), Chest (5)	7	*T. rubrum* (6), *M. canis* (1)	
		Pyoderma gangrenosum-like	Trunk (6), Limbs (28)	34	*T. mentagrophytes* (15), *T. rubrum* (2), *T. erinacei* (2), *E. floccosum* (1), *M. canis* (13), *M. gypseum* (1)	
		Depigmentation with scaling or scaly achromic patches or vitiligo-like	Buttocks+ Limbs	15	*T. rubrum* (3), *M. canis* (11), *E. floccosum* (1)	
		Lichen simplex chronic-like or Lichen ruber planus- like	Limbs+ Trunk	2	*T. rubrum* (1), *M. gypseum* (1)	
		Purpura-like	Limbs (4), Trunk (1), Buttock (1)	6	*T. rubrum* (4), *M. canis* (1), *E. floccosum* (1)	
		Scleroderma-like	Face	2	*T. rubrum* (2)	

ND: Not detected, M: Male, F: Female. *In studies with more than 10 patients, the average age is mentioned.

## Results and discussion

Based on the findings, the average age of patients was 38.56 years old (age range of 2-89 years old according to Table 1, a total of 672 cases). It should be noted that TI commonly targets adults. Some academic dermatology centers have reported 5-10% new atypical chronic cases every year [ 5 ]. In 2018, Stringer et al. showed that children make up 35% of TI cases, and *T. tonsurans* was the most common agent among pediatric TI [ 12 ]. However, in the present review, 2.82% of the cases were children (19 out of 672), and *M. gypseum* was the most common causative agent in children followed by *T. mentagrophytes*. Zoophilic contributing agents are more common in children due to their relationship with household animals; moreover, *T. mentagrophytes* and *M. canis* were other common strains in children [ 13 ]. 

The prevalence of dermatophyte infections depends on the geographical situation [ 14 ]. In recent years, during the COVID-19 pandemic, facial masking in children prepared optimal microenvironments of increased temperature, moisture, and friction on the skin, which could increase the chance of dermatophyte infection. There are three cases of pediatric TI, which were misdiagnosed as allergic contact dermatitis during the COVID-19 pandemic [ 15 ].

### Literature review of the tinea incognita cases

Tinea incognita plaques are more inflamed, less scaly, and with less raised margins, compared to typical tinea infections. Tinea incognita can spread quicker than typical tinea infection [ 8 ]. In addition, due to the lack of proper immune-system response in TI, there is no typical central clearing in TI, unlike the typical tinea infection. In children, TI may be misdiagnosed with pediatric cutaneous diseases, like atopic dermatitis [ 12 ]. 

**Figure 2:**
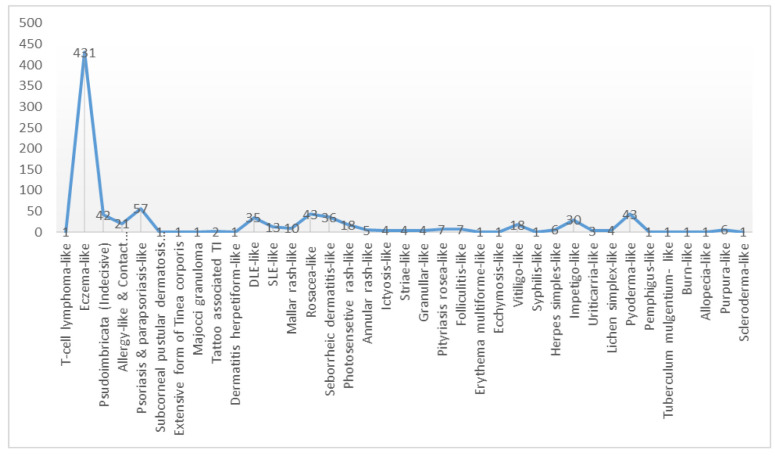
Different patterns of tinea incognita from 2006 to 2023

Figure 2 illustrates the distribution of different patterns of TI according to the present review of articles from the beginning of 2006 to Feb 01, 2023. As revealed in Figure 2, the eczema-like lesion (49.71%, 431 out of 867 cases) was the most common pattern of TI as mentioned in most of the previous studies [ 1 , 16 ]. It is followed by psoriasis (6.57%), pyoderma (4.95%), rosacea (4.95%), and seborrheic dermatitis (4.15%) (Figure 2).

According to Figure 2, rare forms of TI include Zoster-like [ 14 ], pustular psoriasis, dermatitis herpetiform [ 17 ], erythematosus pemphigoid-like [ 18 ], subcorneal pustular dermatosis [ 19 ], Majocci granuloma [ 20 ], Scutula- like lesion and blepharo-ciliaris [ 4 ], parapsoriasis [ 21 ], syphilis- like [ 22 ], and pseudoimbricata [ 23 ].

Tinea pseudoimbricata or tinea indecisiva [ 23 ] is a special subset of TI that mimics tinea imbricata, which is characterized by multiple concentric rings or in other words “ring- within- a- ring’’ lesions [ 24 ]. However, despite tinea imbricata, *Trichophyton concentricum* is not its causative agent. The most common species in tinea pseudoimbricata are *T. mentagrophytes* complex, followed by *T. tonsurans*. The rings in tinea pseudoimbricata are the result of fungal invasion and then suppression of local immunity by intermittent usage of immunosuppressive drugs [ 25 ]. Zoophilic and rare causative agents usually are responsible for rare clinical forms of TI, like *T. mentagrophytes* [ 14 ], *M. audouinii* var *langeronii* [ 19 ], *T. erinacei* [ 20 ], *T. Schoenleinii* [ 21 ]. More usual patterns include Eczema-like, allergy-like [ 26 ], and rosacea-like patterns [ 27 ]. 

Considering the exception of an improper immune system, *T. rubrum* usually causes mild lesions with little inflammation and slight symptoms, which makes it easier to be misdiagnosed. The most common presentation of TI caused by *T. rubrum* is the eczema-like presentation [ 1 , 16 ], even though there are cases with pustular inflammatory skin lesions [ 28 ] and more extensive infections caused by *T. rubrum* [ 29 ]. *Trichophyton rubrum* could invade hair follicles and shafts and make keratinocytes produce interleukin 8 to induce pustules [ 8 ]. In a review performed in 2017 by Dogra et al., *T. rubrum* was the contributing agent for the dissimilar infrequent clinical type of TI-like, eczema/psoriasis-like, impetigo-like, deep dermal infection/ granuloma, molluscum-like, purpuric tinea corporis, cystic granuloma trichophyticum, tinea pseudo imbricata, bullous lesion, pseudomembranous-like, pustular lesion [ 4 ], and nummular scaly papules [ 30 ]. 

More extensive cases were reported when they were found to be neglected, especially in zoophilic strains and immunocompromised patients. In case reports of widespread TI, *T. mentagrophytes*, is commonly the causative agent and these patients are usually under improper treatment with steroids [ 31 , 32 ]. Zoophilic strains are more antigenic to humans, usually cause severe inflammatory reactions, and tend to form pustules in comparison with anthropophilic strains [ 8 , 14 ].

The site of infection varies in different regions of the world, possibly due to different occupations and climatic variations [ 33 ]. According to Dutta et al., the most common site of TI involvement was the face (51%) [ 34 ]. However, Stringer et al. found that face involvement prevalence was equal to that of truncal involvement. In the present review, it was noticed that 42.85% of the total 882 cases in all studies were tinea corporis followed by tinea facie (30.49%) and tinea cruris (5.10%). Stringer et al. found a relationship between gender and lesion sites; they noticed an association between female gender and facial symptoms and male gender and truncal symptoms [ 12 ]. 

### Etiology

According to previous studies, TI was the most common (36%) cutaneous noted adverse drug reaction [ 35 ] and the most common reason for using topical corticosteroids (TCs) was undiagnosed tinea (76.7%); other indications include acne, skin lightening, and melasma. [ 36 ]. Clobetasol propionate and betamethasone topical cream were the most common drugs used alone or in combination with other drugs, such as antifungals and antibacterials [ 33 , 36 ].

In a survey about drug adverse effects, 44.21% of complications were related to topical corticosteroids [ 36 ]. Tinea and TI (13.61%) were not the most common adverse effects in the aforementioned survey, but they were seen objectively among the corticosteroid users. It should be noted that 27.8-87.8% of patients acquired these drugs without a prescription [ 36 - 38 ] and usually based on the recommendation of their friends and relatives [ 38 , 39 ]. In comparison with other studies, TCs were prescribed for 44-78% of patients by a pharmacist or chemist [ 34 ], which had the most prescriptions for tinea-induced corticosteroids [ 34 , 37 , 40 , 41 ]. Although misdiagnosis of TI by general practitioners (GPs) in 21-27.8% of cases [ 37 , 38 ] and dermatologists in 12.5-40.6% of cases [ 33 , 37 , 42 ] had partially led to steroid prescription.

In a study, most of the TI patients were male (71.5%) [ 33 ]; however, in another study, males and females were equally affected (49.7 % male, 50.3% female) [ 37 ]. In addition, females made up most of the cases (61.9%) in another research [ 43 ]. It seems that the usage of TCs is not related to education [ 37 ] and patients with a high school education or more were the most affected group [ 33 , 37 ]. Nevertheless, there may be a relationship between TI and occupation but it is not clear; in a study, housewives (25.3%) and unemployed or retired individuals (20.7%) were the most affected population [ 16 ]. Conversely, in another research, students (39.5%) and government employees (25.5%) were the most involved groups [ 33 ]. 

Tinea incognita can be the result of ringworms, but due to the high prevalence of tinea corporis, TI cases that originated from tinea corporis are more frequent [ 1 ]. Besides, tinea faciei has the least frequency among dermatophytosis; however, due to the misdiagnosis of tinea faciei, it has the highest chance of turning to TI infection among dermatophytosis [ 34 ]. According to Romeo et al., 36% of tinea faciei cases are misidentified and do not receive suitable cure, which may lead to TI [ 44 ].

In tinea incognita faciei, autoinfection from pre-existing tinea of other sites should be considered and mycological diagnosis of different body sites should be performed, especially when *T. rubrum* is responsible for the infection [ 45 ]. There are case reports of tinea faciei incognito, which followed pre-existing onychomycosis. *Trichophyton rubrum* was the causative agent obtained from a case of tinea faciei incognito with a history of onychomycosis of the foot and another with tinea pedis and a history of tinea unguium [ 45 ]. Several researchers have claimed that tattoo inoculation could be a causative factor in the development of tattoo-associated tinea; however, in some cases, tattoo-associated systemic fungal infection may be coincidental [ 46 ].

### Diagnosis

As with every medical diagnosis, the first step to an accurate diagnosis of TI is obtaining a clinical history. There are usually common points among TI patients, for instance, they usually use immunosuppressive treatments, the lesions have mainly an eczema-like shape, appear in the face or trunk, and are unresponsive to steroids [ 8 ]. A mycological examination allows a diagnosis to be made on direct examination and therefore, to start treatment, as culture requires a maximum of 3 weeks in 99% of cases. Powdery, cottony, and granular colonies with a yellowish color on the reverse side and brown pigmentation on the back resemble *T. mentagrophytes* infection [ 27 , 42 ].

*Trichophyton rubrum* usually forms localized eczema-like lesions, which are mostly diagnosed by KOH mount. Lesions caused by *T. mentagrophytes* are usually without or with less scale. It is diagnosed by culture and usually needs systemic antifungal therapy due to disseminated disease or follicular involvement [ 47 ]. *Microsporum* spp. is zoophilic and tends to form more inflamed and pustular lesions, and systemic antifungal therapy is usually necessary for this group [ 8 ]. In some cases, KOH direct microscopy may be negative, and biopsy or histopathology can help detect the fungal infection [ 48 ]. Biopsy and confocal microscopy are indeed diagnostic aids but do not specify the genus or species of the fungus.

Dermoscopy has recently been introduced into the clinical examination of TI. It is helpful in clinics, especially in cases with non-glabrous skin, comma, corkscrew, and Morse code-like hairs [ 49 , 50 ]. Patchy erythema, dotted vessels with peripheral distribution and scattered telangiectasias, white scales with peripheral distribution, perifollicular casts, and transparent thin hairs with comma and cork-screw and morse-code view were seen in dermoscopy [ 51 , 52 ].

Confocal laser scanning microscopy (CLSM) is a non-invasive method, which facilitates the visual detection of superficial fungal hyphae and spores. The CLSM produces high-resolution imaging by reflectance confocal microscopy (RCM) or immunofluorescence following laser illumination of the tissue. Wavelength used in device reports; RCM (830 nm), infrared CLSM (1064 nm), and dual RCM/CLSM (488 nm and 785 nm) [ 53 ]. The RCM could visualize fungal hyphae and spores [ 54 ], but it is unable to differentiate fungal species. Therefore, fungal culture remains mandatory in cases where species identification is important for diagnostic or therapeutic purposes [ 55 ].

The tinea diagnosis method is still controversial, but generally, advanced tests (like a biopsy, histopathology, and mycological examination) are recommended when an old skin disease appears with a new face. In some cases, KOH direct microscopy may be negative, and biopsy and histopathology can help see the fungal infection [ 48 ]. In addition to conventional dermatophyte diagnostics, i.e. direct microscopy and cultivation with subsequent morphological differentiation, molecular methods are becoming increasingly important. 

It has been declared in several studies that the polymerase chain reaction (PCR) method is more sensitive than cultures. Moreover, in some samples, like nails, skin, and hair, it is rarely possible to be cultivated which may lead to false negative results [ 56 ]. Besides this, culture takes at least 4 weeks to display the result, which may lead to misdiagnosis in this period. In addition, PCR is able to detect dead cells as well as dormant cells, which are unable to be cultured due to their silent division activity [ 57 ].

Although there are subjects that need to be proven about the PCR method, it is considered a reasonable method in treatment control [ 57 ]. In PCR, ribosomal genes, such as 18S rDNA and internal transcribed spacer 2 (ITS2) and ITS1 are used in fungal differentiation. The ITS region is preferred in dermatophytes due to the high phylogenic similarity in other regions. There are a few more genes, which are used besides ITS, including chitinase-1 (CHS1), topoisomerase-2 (TOP2), β-tubulin (BTU), or translation elongation factor 1-α (EF-1-α).

Researchers have analyzed the CHS1 gene in different dermatophyte species with different results in the sequences [ 58 - 60 ], but except for *T. rubrum* and *T. violaceum*, none of the closely related species’ groups have been analyzed [ 9 ]. Hence, the CHS1 gene is recommended for the detection of dermatophytes in nail, hair, and skin samples by some scientists [ 61 - 63 ]. Similarly, the TOP2 gene can be used for dermatophyte detection, but it cannot differentiate between the *T. mentagrophytes* species complex isolates [ 64 ]. 

The BTU gene is barely used instead of ITS despite its diversity in Microsporum, since the *Trichophyton* Species are less diverse as there are no differences between *T. benhamiae* and *T. concentricum*, and only one SNP (single nucleotide polymorphisms) between *T. Schoenleinii* and *T. quinckeanum* and two SNP between *T. rubrum* and *T. violaceum* [ 65 ]. Recently, the EF1-α gene has been introduced as an identification gene sequence as precise as ITS in dermatophytes detection. In some species, such as *T. tonsurans* and *T. equinum* or *M. canis* and *M. ferrugineum* EF1-α gene is more specific and useful. [ 66 , 67 ]. 

Molecular methods, like PCR and matrix-assisted laser desorption ionization: time of flight/mass spectrometry (MALDI-TOF-MS) are gaining more importance along with usual dermatophyte diagnostics. The MALDI-TOF-MS is based on the analysis of ionized fragments of biomolecules like peptides, lipids, and saccharides [ 57 ]. Table 2 defines every method for facing dermatophytosis. 

**Table 2:** Methods of dermatophytosis diagnosis

Methods (Brasch 2014) [ 80 ]		Type of Detection	Comment
1	Clinical history and examination	Visual detection	The essential first step of any diagnosis
2	Ultraviolet fluorescence excitation imaging (Wood’s lamp test)	Visual detection	Helpful to screen for Microsporum infection
3	Dermatoscopy	Visual detection	Non-invasive. Rule out non-dermatophyte mycosis
			Visual detection. Tinea capitis = “ comma hairs” or “ C” [ 8 ].
			Morse-code hairs, deformable hairs, translucent hairs, comma and corkscrew hairs, and perifollicular are signs of TI [ 52 ].
4	Confocal laser scanning microscopy (CLSM)	Visual detection	Non-invasive. Visual detection of superficial fungal hyphae and spores. CLSM produces high-resolution imaging by reflectance confocal microscopy (RCM) or immunofluorescence following laser illumination of the tissue.
			RCM has a sensitivity of 52.9–91.7%, a specificity of 57.58–90.2%, a positive predictive value of 61.1–88.6% and a negative predictive value of 68.0–90.5% [ 54 ].
			Wavelength used in devices reports; RCM (830-nm), infrared CLSM (1064-nm), and dual RCM/CLSM (488-nm and 785-nm) [ 53 ].
5	Optical coherence tomography	Visual detection	Visual detection of superficially located fungi
6	KOH mounts (with modifications and refinements) Or direct mycology with KOH	Visual detection	Highly recommended: a very fast and cheap method to detect fungi in stratum corneum, nails, and hair
7	Histology (various staining methods, such as Periodic acid–Schiff stain staining)	Visual detection	Helpful in cases of invasive mycoses and pretreated infections
8	Culture (on various agars)	Organism detection	The conventional method to identify fungi is time-consuming (it takes weeks and may lead to delay in treatment ) and dependent on the vitality of fungi [ 80 ]. Culture is the gold standard for the diagnosis of dermatophytosis [ 55 ].
9	Genetic methods (polymerase chain reaction-based analyses)	Organism detection	Fast technique to detect and identify even non-vital fungi; awaits standardization; special equipment needed
10	Matrix-assisted laser desorption ionization–time-of-flight analysis	Organism detection	Fast technique to identify cultured fungi; based on organism fragments detection; awaits standardization; sophisticated equipment needed

### Treatment

As it was mentioned before, since the most common cause of TI is corticosteroid abuse followed by misdiagnosis and improper treatment, the most important step in TI treatment is ceasing the corticosteroids. The next steps depend on the overall situation of the patient to prescribe the most suitable antifungal treatment. In recalcitrant cases, systemic antifungals are prescribed the same as in hair and nail involvement systemically and topically; Usually terbinafine or itraconazole along with clotrimazole is prescribed [ 47 ]. Nowadays due to the new emerging terbinafine-resistance isolates, specialists prefer to prescribe azoles instead of terbinafine including itraconazole [ 68 ] or voriconazole [ 69 ].

## Conclusion

Based on the review of the literature, a corticosteroid may cause different clinical manifestations of dermatophytes that might be very different from those that are commonly described. Dermatophytosis is a highly communicable disease and there is a concern about the transmission of antifungal-resistant species. Consequently, dermatologists should obtain the full medical history of the patients to make correct diagnoses. It is also of great importance to encourage an exact identification of the etiological agent in some uncertain cases. 

To conclude, the prescription of topical/oral corticosteroids should still be limited and done with caution until confirmation of diagnosis, particularly considering the use of steroids during the COVID-19 pandemic. Furthermore, the knowledge of skilled mycologists can play a key role and make a difference in changing the scientific community. This review highlighted the importance of mycological tests in the case of atypical skin lesions to choose appropriate treatment and avoid the spread of drug-resistant species.
